# Correction of severe upper-eyelid sunken deformity in Asians by double-eyelid procedure and combination of orbital fat pad repositioning and autologous fat transplantation: a one-year follow-up study of 79 cases

**DOI:** 10.3389/fsurg.2024.1358600

**Published:** 2024-05-01

**Authors:** Haiyan Shen, Jufang Zhang, Weiqiang Tan, Xiujun Cai

**Affiliations:** ^1^ Department of Plastic Surgery, Zhejiang University School of Medicine, Hangzhou, China; ^2^ Department of Plastic Surgery, Hangzhou First People's Hospital, Hangzhou, China; ^3^ Department of Plastic Surgery, Sir Run Run Shaw Hospital, Zhejiang University School of Medicine, Hangzhou, China; ^4^ Department of General Surgery, Sir Run Run Shaw Hospital, Zhejiang University School of Medicine, Hangzhou, China

**Keywords:** upper-eyelid sunken deformity, double-eyelid procedure, autologous fat transplantation, orbital fat pad repositioning, follow-up study

## Abstract

**Background:**

Nowadays, people's pace of life continues to rapid up, and many bad habits will accelerate the aging of the eye periphery, and patients with sunken upper eyelids are to be found in younger people. In young Asians, single eyelids are often accompanied by upper eyelid depression, so correcting the upper eyelid depression during blepharoplasty becomes a higher challenge for plastic surgeons. Current surgical methods for upper eyelid depression include three major categories: tissue repositioning, injection and filling, and combined use. According to grades 1 and 2 are mild or moderate upper eyelid sunken. The sunken can be well corrected only by repositioning the orbital fat pad, while the correction effect for severe upper eyelid sunken in grades 3 and 4 is Poor, need to be used in combination to achieve the desired effect.

**Purpose:**

The authors sought to determine whether, for patients with single eyelids and severe upper eyelid depression of grades 3 and 4, combined with orbital fat pad repositioning and autologous fat transplantation during blepharoplasty, an aesthetic and youthful blepharoplasty can be achieved.

**Methods:**

This study included 79 patients with single eyelids and severe upper eyelid depression of grades 3 and 4 who received treatment between June 2020 and July 2022. All patients underwent double eyelid surgery plus orbital fat repositioning and autologous fat grafting.

**Results:**

After a minimum follow-up period of 1 year, overall patient satisfaction was 92%. The recurrence rate within the first year was 6% and the complication rate was 5%.

**Conclusion:**

This combined surgery may be an option for young Asians with single eyelids and severe upper eyelid depression. In this study, the surgery resulted in natural-looking double eyelids and younger-looking eye sockets in most patients. A combination of different surgical methods based on the patient's preoperative condition is critical to achieving long-term correction.

## 1 Introduction

Double eyelid surgery is the most common cosmetic surgery among young people in Asia. However, for patients with single eyelids and upper eyelid depression, double eyelid surgery is required to correct the upper eyelid depression at the same time to obtain satisfactory results. Upper eyelid depression refers to the inward collapse of the area between the upper tarsal plate and the infraorbital rim, which often occurs simultaneously with eyelid skin laxity and ptosis (1). An obviously sunken upper eyelid will affect the aesthetics of the eyes and give people the impression of fatigue and aging. It will also cause symptoms such as visual fatigue and dry eyes. In order to rejuvenate the periorbital area, there are endless ways to improve upper eyelid depression, each with its own advantages and disadvantages. They mainly include three categories: tissue repositioning, injection and filling, and combined application. In clinical work, it is particularly important to provide patients with personalized treatment plans for different degrees of upper eyelid depression. For mild or moderate upper eyelid depression, the depression can be well corrected only by repositioning the orbital fat pad, but it is more difficult to correct severe upper eyelid depression. For this type of patients with single eyelids who need both double blepharoplasty and correction of upper eyelid depression, we performed double eyelid surgery on 79 patients with this type of patients, combined with orbital fat pad repositioning and autologous fat transplantation, and followed up for one Year later, it has been proven that it can achieve the desired results.

## 2 Materials and methods

### 2.1 General information

To classify upper eyelid depression according to the Park classification standard, the patient sits and looks straight ahead, and the horizontal distance from the upper orbital edge to the most depressed upper eyelid is measured. Grade 1: depression depth ≤0.5 cm; grade 2: 0.5 cm < depression depth <1 cm; grade 3: depression depth ≥1 cm; grade 4: depression depth the same as grade 3, accompanied by ptosis (1). We clinically define grade 1 and 2 upper eyelid dents as mild to moderate upper eyelid dents, while grade 3 and 4 upper eyelid dents are classified as severe upper eyelid dents.

This study selected 79 patients with single eyelids and severe upper eyelid depression, aged 25–46 years old. All patients were female, including 69 patients with grade 3 upper eyelid depression and 10 patients with grade 4 upper eyelid depression. Most cases were bilateral (77 cases), only 2 cases had unilateral severe ptosis. All patients with grade 3 upper eyelid dents underwent upper eyelid fat flap transfer and repositioning combined with autologous fat graft injection to fill the upper eyelids between June 2020 and July 2022. Patients with grade 4 upper eyelid dents also underwent simultaneous ptosis correction surgery followed up for 1 year after surgery. All patients in this study had informed consent, which was approved by the hospital ethics committee.

## 3 Surgical method

### 3.1 Blepharoplasty

Design the double eyelid line according to the patient's own conditions and the degree of upper eyelid depression, mark the location and range of the upper eyelid depression, and evaluate the amount of fat that needs to be filled in the upper eyelid to obtain better surgical results. All patients underwent blepharoplasty. The skin was peeled in sequence according to the designed double eyelid line, and the orbicularis oculi muscle and roof layer were incised to fully expose the upper eyelid orbital septum and open the orbital septum. Due to severe upper eyelid depression, The patient's upper eyelid tissue is thin and the upper eyelid tissue is completely preserved. The orbicularis oculi muscle at the lower edge of the incision, the levator palpebrae superioris aponeurosis, and the orbital septum stump are sutured with 7-0 nylon suture to form a physiological double eyelid.

For patients with blepharoptosis, depending on the cause and degree of ptosis, the surgical method of shortening and advancing the levator aponeurosis or shortening and advancing the levator aponeurosis combined with Muller muscle can be selected to correct ptosis.

### 3.2 Orbital septum fat flap transfer and reset

In patients with severe upper eyelid sunkenness, the fat position shifts upward and inward, and there are often thick fibrous adhesions around it. Only by fully loosening these adhesions can the fat mass in the orbital septum be completely released and prevent it from retracting again. The upper eyelid Orbital fat in patients with severe upper eyelid depression is often very thin in the medial and middle. Sometimes only the fat capsule is visible, but there is still some capacity in the lateral extension of the central fat mass. Therefore, the pedicle of the lateral extension is often partially cut off to It can move freely, and then use the central fat mass as the pedicle to transfer the lateral extension to the medial side and fix it on the upper edge of the tarsal plate, which can better improve the medial depression. At this time, the double eyelids are formed and part of the upper eyelid depression is improved through the release of orbital fat mass. The amount of fat that needs to be filled can be assessed based on the patient's condition, Figure 1.

**Figure 1:**
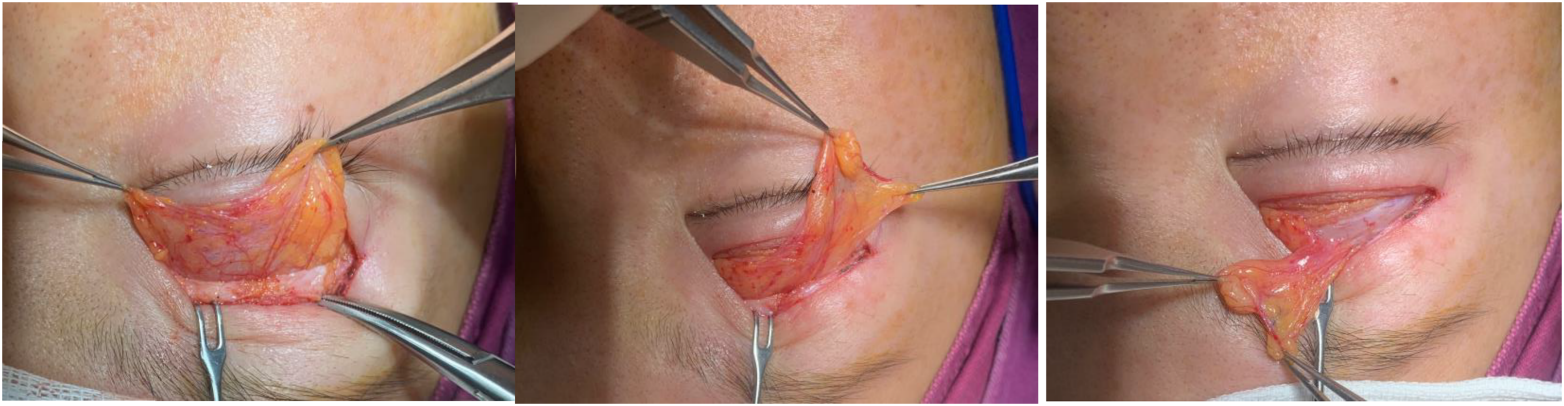
Transfer the lateral extension of the upper eyelid orbital fat to the medial side using the central fat mass as the pedicle.

### 3.3 Autologous fat transplantation

After the orbital septum fat is released, the upper eyelid depression is relieved. The first fat injection volume is 1–3 ml, so the amount of fat that needs to be suctioned is about 50 ml. For patients who need secondary fat injection after evaluation, an extra portion of fat can be suctioned, 80–100 ml of fat is sufficient.

The liposuction site is the abdomen or inner thighs. First, the surgical area is swollen and anesthetized, and 100–200 ml of swelling fluid is injected into the subcutaneous fat layer. Wait 20 min. Fat was harvested and processed according to (2). In brief, a suction needle (diameter 2.5 mm) connected to a 10-ml syringe was inserted into the fat layer of the abdomen/inner thigh. The plunger was pulled to 4–5 ml scale to create a low vacuum pressure. By repeated back and forth movement of the 10-ml syringe in a “spike of a wheel “pattern, the fat particle was harvested. Simultaneously, the other hand was placed over the harvesting area to feel and control the suction direction and depth. The 10-ml syringe, full of fat and fluid, was removed and placed vertically for several minutes.

Once the upper fat layer was separated from the lower tumescent fluid layer in the syringe, the lower fluid was discarded. Then 15–20 ml of the upper fat was collected. The fat was divided equally into two 10-ml syringes closed with a blunt cap and centrifuged at 1,500 g for 3 min. The centrifuged syringe was then removed from the holder, and the lower fluid layer discarded. The superficial liquid fat layer was absorbed by the gauge or cotton swab. A concentrated and purified fat particle was achieved. Using a transfer adapter, the purified fat particle in the 10-ml syringe was transferred into individual 1-ml syringes. The blunt tip cannula (diameter: 1.0 mm) with a side port was attached to the 1-ml syringe ready for injection.

Upper eyelid fat injection is performed using supraorbital nerve block. We select the middle eyebrow and the lateral canthus of the zygomatic arch as the needle insertion points. 1 ml syringe is generally used during injection to reduce fat cell damage. Since the orbital septum fat flap is fully released, the space between the superior orbital rim and the eyeball has been filled. We choose the suborbicularis oculi muscle layer (ROOF) as the fat injection level. The purified granular fat is multi-tunneled, tension-free, and evenly injected into the superficial and deep surfaces of the ROOF layer. The amount of injection should be approximately 20% over the upper eyelid. Mild swelling, average 1.0–2.0 ml per side. Since the upper eyelid skin and orbicularis oculi muscle of patients with severe upper eyelid ptosis are very thin, try to avoid injecting fat particles into the subcutaneous and orbicularis oculi muscle layers to avoid causing uneven upper eyelids and bloated upper eyelids. If the orbital fat flap is very thin, a small amount of fat particles can be injected into the orbital fat capsule to restore the volume without affecting the function of the levator palpebrae superioris muscle. Although the absorption rate after free fat tissue transplantation is as high as 45%–60%, the orbital septum fat flap reset to the medial side has a rich blood supply and a constant volume in the long term, so fewer patients require secondary fat filling, the operation diagram of fat transfer is shown in Figure 2.

**Figure 2:**
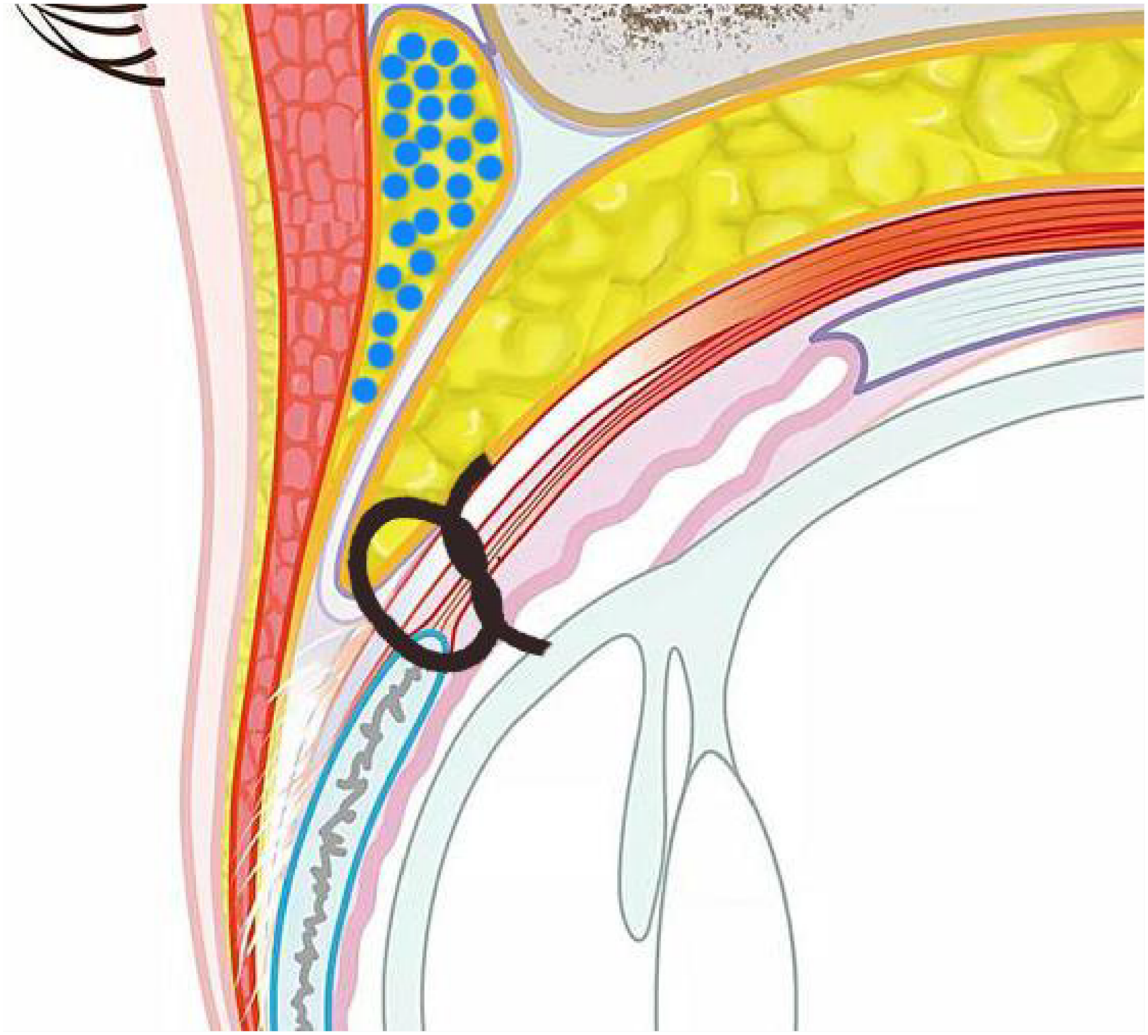
The round blue dots represents fat layer of injection (under layer of roof). The orbital septum fat is released and fixed to the upper edge of the tarsal plate.

For a small number of patients who are found to have serious insufficient capacity of the orbital septum fat flap during the operation, we will consider a second-stage fat gel transplantation, extracting about 100 ml of fat, and after injecting fat particles in the first stage, the remaining fat will be prepared into fat gel (SVF-gel). After cryopreservation, the second injection is usually performed after 3–4 months. The additional injection volume depends on the patient's upper eyelid fat absorption, with an average of 1.0–2.0 ml per side. Preparation process of fat gel (SVF-gel): (1) Centrifuge the extracted fat particles at 1,500 g for 3 min, discard the lower liquid, retain the upper fat, and transfer the upper fat and middle high-density fat (Colemn fat) to a syringe. (2) Pass two 10 ml syringes containing adipose tissue through the fat converter, squeeze and inject repeatedly at a speed of 10–20 ml/s, and inject continuously back and forth until the fat particles are completely emulsified. (3) Filter the chyloform adipose tissue to remove larger fat cells and thick fiber cords in the tissue, seal it into the syringe, pull back the syringe plug, and maintain a certain negative pressure in the syringe. The mixture was centrifuged twice at 2,000 g for 3 min. (5) After centrifugation, the mixture is divided into 3 layers. The lower layer is the swelling liquid layer, the upper layer is the oil layer, and the middle layer is the desired product SVF-gel, which is the autologous fat grafting material of highly concentrated ADSCs and extracellular matrix. Transfer the obtained SVF-gel into a 5 ml syringe, seal and freeze for later use (3).

Sutures were removed 7 days after double eyelid surgery, and patients underwent routine follow-up at 1 month, 3 months, 6 months, and 1 year to evaluate the aesthetic results and whether secondary SVF-gel filling was needed.

## 4 Results

### 4.1 Surgical repair effect

The sutures were removed one week after the operation. The upper eyelids were slightly swollen and bruised, and the double eyelid line curved naturally. One month after surgery, the swelling subsided significantly and the upper eyelid became plump. The width, curvature and upper eyelid fullness of the double eyelids all tended to be stable three months after surgery. There were 79 cases of grade 3 or 4 upper eyelid depression, 70 of which had no upper eyelid depression; 7 cases improved to grade 1; 2 cases had grade 2. Both cases received a second supplementary injection of SVF-gel 3 months after the first surgery, with an injection volume of 1.0–2.0 ml on each side. Both cases achieved a plump appearance of the upper eyelids during the six-month postoperative follow-up. One year after the operation, the width and curvature of the double eyelids were natural and smooth, and the fullness of the upper eyelids: the upper eyelids were restored to fullness in 70 cases and grade 1 depression in 9 cases.

### 4.2 postoperative complications

Mild swelling and bruising occurred in the early stage, which resolved within 2 weeks. There was no postoperative double eyelid detachment, triple eyelids, incomplete eyelid closure, or secondary ptosis. No liquefaction or nodules occurred after fat injection into the upper eyelid. The upper eyelid does not have a bumpy appearance.

### 4.3 Patient satisfaction after surgery

The patients’ self-evaluation at the 1-year follow-up included: double eyelid curvature, upper eyelid fullness, and facial rejuvenation. The evaluation is 95% satisfactory and 5% acceptable.

### 4.4 Typical cases

Case 1: A 30-year-old woman had grade 3 upper eyelid depression with a depth of 1cm. According to the patient's requirements for repairing the upper eyelid depression, orbital septum fat repositioning surgery combined with upper eyelid free fat injection filling was performed. The injection dose was 1 ml on the left side and 1.2 ml on the right side. Six months after the operation, the upper eyelids were plump, the double eyelid line was smooth, the eyelid skin was smooth, the double eyelids were symmetrical, the wound was hidden, there were no complications, and the patient's satisfaction was high see Figure 3.

**Figure 3:**
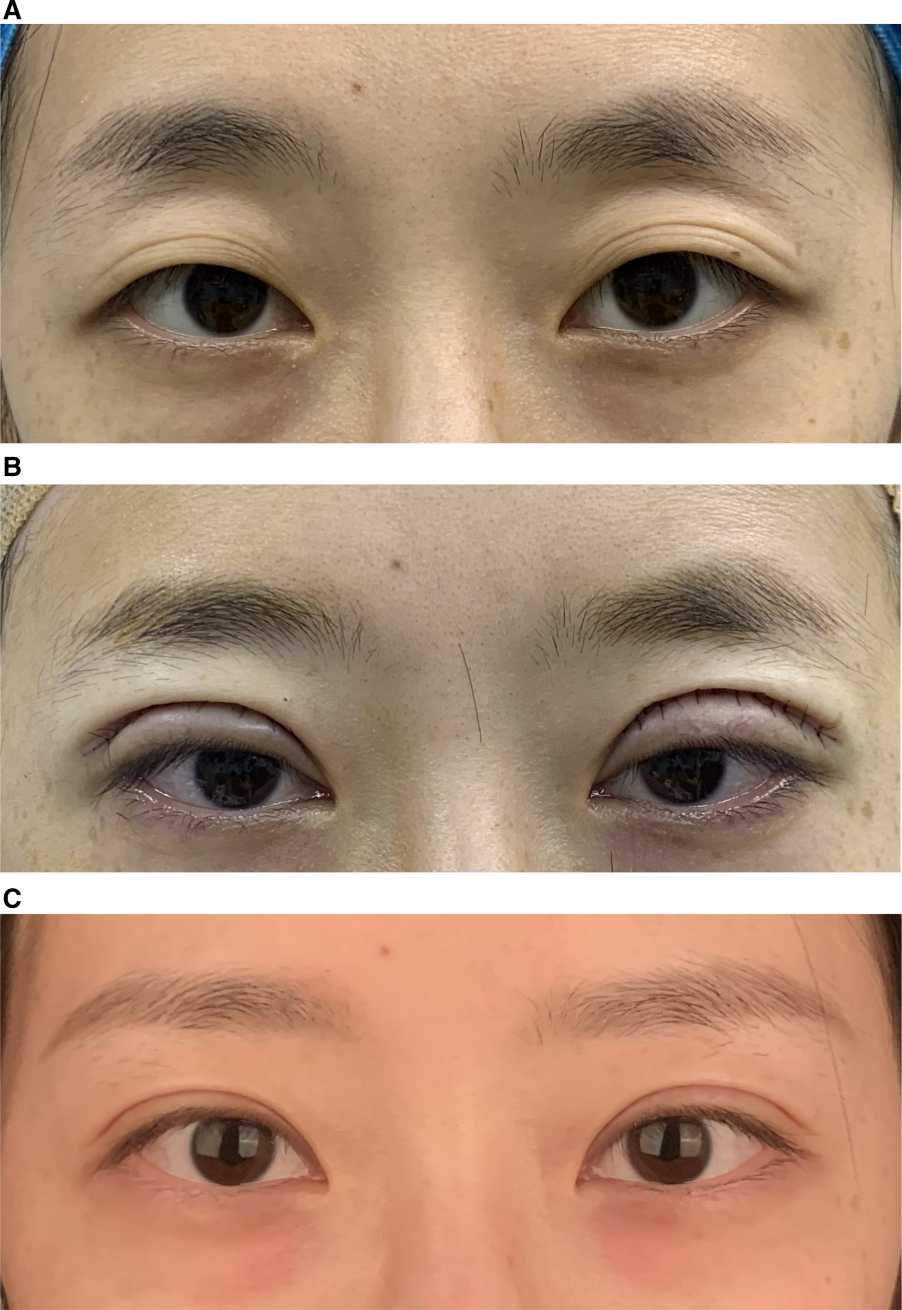
(**A**) Pre-operation. (**B**) Immediately post-operation. (**C**) 3 months post-operation.

Case 2: A 36-year-old woman had grade 4 upper eyelid depression with mild ptosis with a depth of 1.1 cm. Double eyelid surgery combined with levator aponeurosis advancement surgery, synchronous orbital septum fat repositioning surgery combined with upper eyelid free fat injection were performed. The injection dose was 1.3 ml on the left side and 1.2 ml on the right side. At the 3-month postoperative follow-up, the upper eyelid was sunken to grade I, the double eyelid line was smooth, the eyelid skin was smooth, and the double eyelids were symmetrical. After communication, the patient requested a supplementary injection of SVF-gel. The dosage of the second supplementary injection was 1.0 ml for the left side and 0.9 ml for the right side. At follow-up 3 months after the second injection (half a year after the first operation), the double eyelids were natural and smooth, the upper eyelids were plump and uneven, and the patient was satisfied see Figure 4.

**Figure 4:**
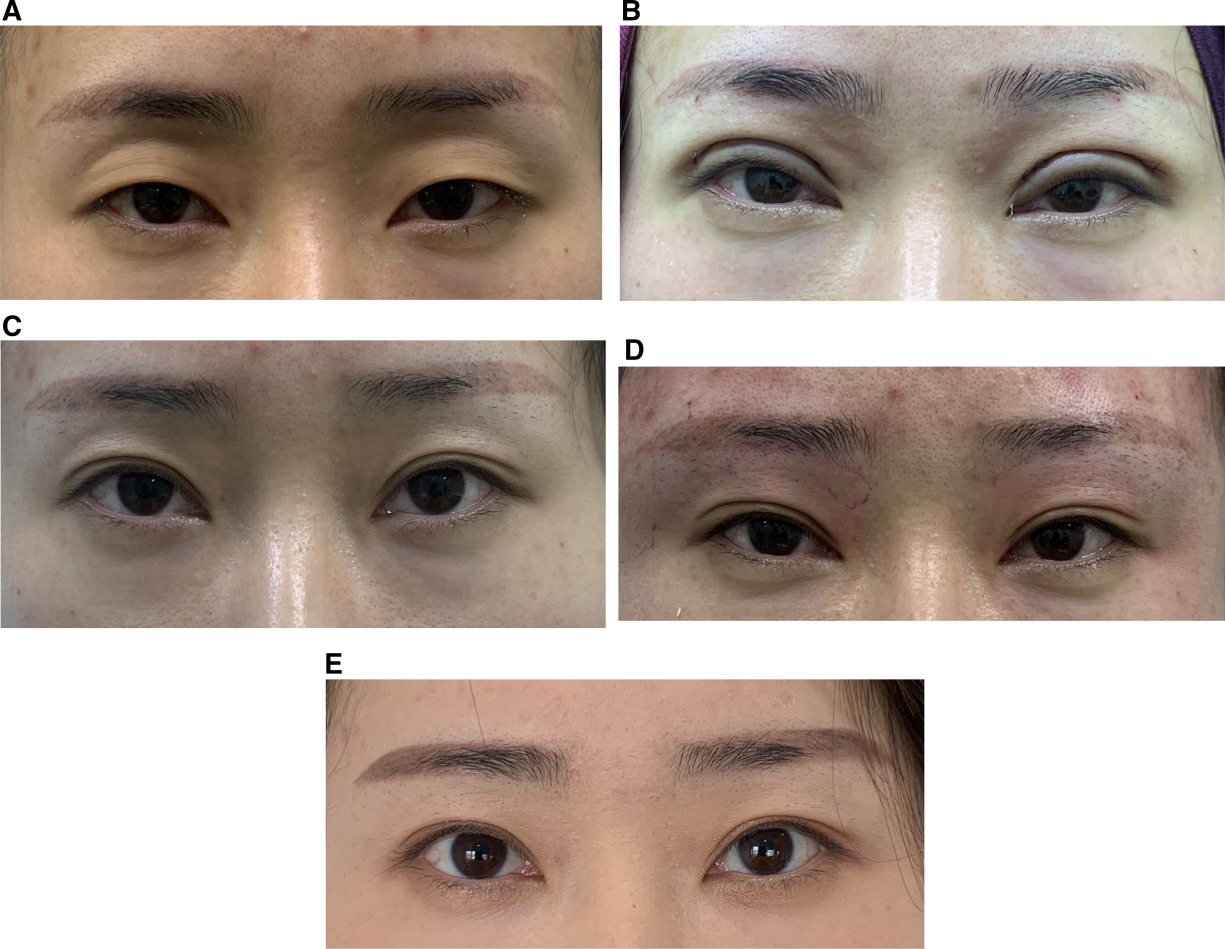
(**A**) Pre-operation. (**B**) Immediately post-operation. (**C**) 3 months post-operation. (**D**) Immediately post SVF-gel injection. (**E**) 6 months post-operation (3 months post-2nd injection).

## 5 Discussion

Sunken upper eyelid refers to a sunken deformity between the lower edge of the eyebrow and the upper edge of the eyeball for whatever reason (1), which often occurs at the same time as eyelid skin laxity and drooping. According to the cause of onset, upper eyelid depression can be divided into congenital and acquired. Congenital upper eyelid depression is mostly caused by lack of fat in the orbital septum or ethnic characteristics, and the anatomical position of the upper eyelid is normal. Acquired upper eyelid depression can be subdivided into aging, surgical and traumatic. Congenital upper eyelid depression often shows that the bony structure of the orbit is prominent, the fat and subcutaneous tissue around the orbit are weak, and the prominent bony structure is disproportionate to the weak soft tissue. At the same time, there is a lack of fat in the orbital septum of the upper eyelid, resulting in an obvious sunken upper part of the eye socket. The eyeballs appear to protrude forward. In some patients with reduced vision and axial length of the eye, the upper eyelids appear particularly sunken. Therefore, patients with congenital upper eyelid depression can show aging orbital characteristics at a very early age.

Europeans and Americans have less fat in the orbital septum than Asians. They have a tight orbital septum and are prone to upper eyelid depression. Acquired upper eyelid depression are mainly divided into senile upper eyelid depression, surgical upper eyelid depression, traumatic upper eyelid depression, and upper eyelid depression caused by acquired chronic diseases. Senile upper eyelid depression is mainly related to the absorption of bony structures and atrophy of soft tissues. Surgical upper eyelid depression is also very common in China. A few years ago, many patients in China requested wide double eyelid surgery. While designing a wider double eyelid line, excessive removal of orbital septal fat will also cause postoperative upper eyelid depression. Traumatic upper eyelid depression is partly caused by the imbalance between the volume of the orbital cavity and the volume of the orbital contents after orbital fractures, and partly caused by the abnormal position of the orbital contents. For different reasons, different surgical methods can be used to better correct upper eyelid depression.

The principle of repairing upper eyelid depression is to fill the tissue and restore the volume and normal anatomical relationship of the orbital septum. At present, there are various correction methods for upper eyelid depression, which can be summarized into three categories: tissue repositioning; injection and filling; and the combined use of multiple methods. Repositioning of tissues: All layers of soft tissue in the upper eyelid and orbital area, including orbital septal fat, orbicularis oculi muscle flap, suborbicularis oculi fat flap ROOF, frontalis fascia-subcutaneous fat flap, and periosteal flap, can all be used Pedicled local transposition to fill the upper eyelid depression. The most widely used and effective method at present is the release and reset of orbital septal fat. In patients with congenital upper eyelid depression, upper eyelid depression mainly occurs on the medial and central sides, especially grade 1 and 2 upper eyelid depression. Many clinicians have discovered during surgery that no matter how deep the upper eyelid is, the lateral extension of the central fat pad always exists, with only a difference in size. Therefore, transferring the lateral extension of the central fat pad to the inside with the central fat pad as the pedicle can effectively fill the medial depression, and there is orbital bone support on the lateral side, so there will be no depression after the fat flap is transferred, but it will reduce lateral sagging and improve the appearance of “triangular eyes”. The pedicled orbital septum fat flap has good blood supply, so there is no problem of absorption.

The fat anatomy of the inner and outer orbital septum is the same, making the effect more natural. This method is simple to operate, has little damage, few complications, fast recovery, and good postoperative results. We found that at one-year follow-up, the effective rate of grade 1 upper eyelid depression, through fat flap reset is more than 90%. Grade 2 can also reach 50%, while grade 3 and 4 upper eyelid depression cannot be effectively corrected only by resetting the fat flap. To achieve good correction results, we will use a combination of multiple methods to achieve better clinical results for these patients with severe upper eyelid depression.

The second major method is tissue filling and injection, which is also the mainstream method for correcting upper eyelid depression. Tissue filling includes the use of synthetic fillers and filling with autologous tissue grafts. When using synthetic fillers, hyaluronic acid can be injected deep into the orbicularis oculi muscle. porous high-density polyethylene, expanded material, etc. can be implanted inside and outside the infraorbital wall to increase the amount of tissue in the upper eyelid area or enhance bony support. These methods have unique advantages in some cases, but they are difficult, high-risk, and have serious complications, resulting in poor patient acceptance and have not been widely used clinically. In addition to the mainstream adipose tissue, autografts also include bone and cartilage. Autologous bone or cartilage has good compatibility and a relatively good survival rate, but they are hard in texture, absorbed slowly, and easily lead to the formation of granulomas. The surgical procedure is complex and there are few clinical applications.

At present, the most common autologous filling tissue is adipose tissue, including block adipose tissue, dermal adipose tissue, fascia-fat composite tissue free transplantation, and autologous fat particle transplantation. However, free transplanted fat blocks have no blood supply, and the fat survival rate is difficult to predict. Ischemia, liquefaction, and necrosis can lead to a low survival rate, but there are also cases where the fat survival rate is too high, resulting in a bloated appearance of the upper eyelids. There is a clinical case report that local fibrosis caused by free fat grafting resulted in scar adhesion and abnormal appearance of the eyelids after surgery. Autologous dermal fat flaps and fascia-fat composite tissue flaps have strong anti-infection and good supporting effects, and can correct various degrees of upper eyelid and facial depression. However, they are relatively difficult to obtain and cause significant postoperative swelling. They are more clinically used for severe upper eyelids depression, orbital area depression after eyeball removal or hemifacial atrophy.

Autologous fat particle injection are currently the most commonly used surgical method to correct upper eyelid depression due to its wide range of materials, simple operation, and minimal invasiveness. An important factor in its higher survival rate than free fat grafting is that fat particles are small in size and can obtain nutrients from tissues in an early ischemic environment in a timely manner, rebuild blood and lymph circulation and regenerate capillaries, thereby improving the survival rate of transplantation. It is generally believed that the smaller the damage to fat cells, the higher the survival rate. Therefore, the fat is centrifuged and purified to remove swelling fluid and necrotic fat cells, and the high-quality fat particles obtained will have a higher survival rate after transplantation. Some scholars have proposed that the optimal centrifugation rate is 1,000 r/min (3 min). After centrifugation, the activity and purity of adipocytes are improved. Many clinicians now use the static method to process fat. This method can shorten the residence time of fat outside the body, reduce the risk of contamination, and preserve the microenvironment of fat cells to the greatest extent. When we process fat suction, we like to centrifuge it at 1500 g for 3 min to remove swelling fluid and damaged fat cells, and take the high-density fat in the middle for injection and transplantation.

The selection of the needle insertion point and injection level of fat grafting is also particularly important for the surgical effect and reducing complications. Because the upper eyelid skin is thin, if the injection is uneven, the layers are too shallow, or the subcutaneous scar is adhered, uneven upper eyelids and asymmetry on both sides may easily occur. Injecting too deeply or injecting too much volume can easily lead to bloated upper eyelids, or compression of the levator palpebrae superioris muscle, causing temporary ptosis. In addition, serious complications such as infection, liquefaction, necrosis, fibrous wrapping, tear gland damage, fat embolism, and blindness can occur.

Tansatit et al. dissected 20 cadavers and conducted ultrasound studies on 30 healthy volunteers. It is proposed to select the needle insertion point between the lateral canthus and the lateral eyebrow. When the injection level is close to the periosteum of the supraorbital rim, it can fill the most natural appearance and reduce the incidence of arterial damage. We will choose the middle part of the upper eyebrow as the needle insertion point to avoid the subcutaneous course of the supraorbital artery and supratrochlear artery. Our consideration is that it is close to the sunken area of the upper eyelid, especially the middle and inner side of the upper eyelid where it is the most sunken, so there will be less damage, the filling effect is good. After the needle is inserted, the blunt needle moves downward close to the bone surface, under the frontalis muscle and orbicularis oculi muscle, and injects fat particles in multiple layers, multiple tunnels, and evenly on the deep and superficial surfaces of the suborbicularis oculi fat (ROOF). after injection, the upper eyelid will be smooth and plump, with a natural shape. When injecting, the amount should be appropriately exceeded by 20% until the upper eyelid is slightly swollen, with an average of 1.0–2.0 ml per side. Some doctors also recommend that the injection should be 1–2 mm higher than the level of the eyebrow arch.

Lin et al. (4) introduced the application of MAFT-Gun (Micro Autologous Fat Transplantation Injection Gun) for more precise and quantitative injections, with an output of 240 times per milliliter of fat. The fat was precisely injected into multiple levels, including suborbicularis oculi fat (ROOF), orbicularis oculi, between the orbicularis oculi muscle and the eyelid dermis, the postoperative results are highly satisfactory. Autologous fat particle injection and transplantation are suitable for correcting different degrees of upper eyelid depression. However, clinical follow-up found that grade 1 and 2 upper eyelid depression have obvious correction effects and high patient satisfaction. Grade 3 and 4 upper eyelid depression can not only be corrected by fat injection. Increasing the amount of transplantation may easily lead to problems such as central necrosis and fibrosis of the transplanted fat, uneven skin, insufficient correction, adhesion of the levator palpebrae superioris muscle, and mild ptosis due to excessive mechanical load. Therefore, for patients with severe grade 3 or 4 upper eyelid depression who require double eyelid surgery, we combine double eyelid surgery, orbital septum fat flap repositioning and upper eyelid fat particle free transplantation to correct it.

The advantages of combined application are: 1. After repositioning of orbital fat flap, it can fill the gap between the upper orbital rim and the eyeball, but fat injection transplantation is difficult to fill this level; 2. Due to the homology of the orbital septum fat flap and There is abundant blood supply, and the long-term volume will not be absorbed. Combining the two methods can not only reduce the amount of fat transplanted, avoid complications caused by large-volume fat transplantation, but also ensure long-term postoperative effects. Among the 79 patients we enrolled, only two cases showed insufficient correction of the upper eyelid sunken in long-term follow-up, and a second supplementary injection of SVF-gel was performed 3 months after surgery.

This study included 10 patients with grade 4 upper eyelid depression and mild to moderate ptosis. Weakness of the levator palpebrae superioris or structural changes in the levator palpebrae superioris aponeurosis, such as elongation, thinning, holes, or partial or complete separation from the tarsal surface, will cause the levator palpebrae superioris muscle and orbital septal fat to shrink and move backward, appearance of widening of upper eyelid folds and upper eyelid depression. Park can improve upper eyelid depression by adjusting the connection between the levator aponeurosis and the tarsal plate, folding and advancing the levator aponeurosis. However, the ability to change the position of the orbital septum fat through the displacement of the levator aponeurosis is limited. It is only suitable for mild upper eyelid depression combined with mild to moderate ptosis. For grade 4 upper eyelid depression, on the basis of correcting the ptosis, we all combined orbital septal fat repositioning and free fat transplantation, and the long-term postoperative results were satisfactory. Only one case underwent a second supplementary injection of SVF-gel 3 months after surgery.

For patients with single eyelids and severe upper eyelid depression, a combination of multiple methods can not only obtain the appearance of smooth and flexible double eyelids, but also correct the upper eyelid depression and restore the youthful appearance of the upper eyelids. For patients with ptosis, it can increase the brightness of the upper eyelids and achieve multiple correction effects; After the orbital septal fat flap is reset, it fills the gap between the upper orbital rim and the eyeball, while it is difficult for fat injection and transplantation to achieve this. It avoids the unnatural appearance of the injected material moving with the movement of the eyeball after free fat filling. Due to the homology of the orbital septum fat flap and its rich blood supply, the long-term volume will not be absorbed. Combining the two methods can not only reduce the amount of fat transplanted, avoid complications caused by large-volume fat transplantation, but also reduce the complications of poor long-term results caused by the absorption of transplanted fat after surgery. When releasing the upper eyelid orbital septum fat, fully loosen the fibrous adhesion between the upper part of fat mass and the roof layer, and the adhesion between the lower part of fat mass and the levator aponeurosis. There are often some thick fibers in this layer. While releasing, the integrity of the levator aponeurosis surface is maintained to avoid iatrogenic ptosis. When resetting orbital septum fat, the pedicle of the lateral extension of the central fat mass is often partially severed until it can move freely. Since the surface of the fat mass is rich in blood vessels, the bleeding can be fully stopped after the separation to avoid local hematoma. When the lateral extension is moved to the medial side using the central fat mass as the pedicle, it is carefully fixed on the upper edge of the tarsal plate and under the roof on the medial side to avoid the recurrence of the medial depression caused by the later movement of the fat mass.After the orbital septum fat is reset, carefully evaluate the amount of free fat needed to be transplanted. Considering that the orbital septum fat has a rich blood supply and a low postoperative absorption rate, a moderate amount of free fat is sufficient to avoid the appearance of bloated upper eyelids in the long term after surgery. When injecting fat into the upper eyelid, focus on the area with the highest degree of depression under the orbital rim as the key injection area, especially the middle and medial sides of the upper eyelid (where triple eyelids are most likely to occur). The outer side should also be taken into consideration but avoid excessive injection volume on the outer side to prevent the occur of droopy (triangular) appearance.

For patients with emaciation, the possibility of low survival rate after fat injection and the need for secondary fat injection should be considered. Part of the fat needs to be suctioned appropriately during the operation to make fat glue for cryopreservation, and 3 months after operation, the patient is asked to follow up to evaluate whether fat injection is needed again to replenish the upper eyelid capacity.

In short, for patients with single eyelids and severe upper eyelid depression of grade 3 or 4, combined with double eyelid surgery, orbital fat flap repositioning and upper eyelid fat particle free transplantation for correction. It can achieve a natural and smooth double eyelid, as well as a full and youthful appearance of the upper eyelid. This surgery is minimally invasive and has good long-term follow-up results, which can be safely promoted and used in clinical patients. However, the possible risk of blindness caused by fat embolism and optic nerve compression should not be ignored. More cases are needed for further research.

## Data Availability

The raw data supporting the conclusions of this article will be made available by the authors, without undue reservation.
